# On the improvement of blood sample collection at clinical laboratories

**DOI:** 10.1186/1472-6963-14-12

**Published:** 2014-01-09

**Authors:** Alex Grasas, Helena Ramalhinho, Luciana S Pessoa, Mauricio GC Resende, Imma Caballé, Nuria Barba

**Affiliations:** 1 Department of Economics and Business, Universitat Pompeu Fabra, Barcelona, Spain; 2 Barcelona GSE, Barcelona, Spain; 3 IN3 Universitat Oberta de Catalunya, c/ Roc Boronat, 117, 08018 Barcelona, Spain; 4 AT&T Labs Research, Florham Park, NJ, USA; 5 Catlab, Barcelona, Spain

**Keywords:** Blood sample transportation, Vehicle routing problem, Genetic algorithm, Operations research

## Abstract

**Background:**

Blood samples are usually collected daily from different collection points, such hospitals and health centers, and transported to a core laboratory for testing. This paper presents a project to improve the collection routes of two of the largest clinical laboratories in Spain. These routes must be designed in a cost-efficient manner while satisfying two important constraints: (i) two-hour time windows between collection and delivery, and (ii) vehicle capacity.

**Methods:**

A heuristic method based on a genetic algorithm has been designed to solve the problem of blood sample collection. The user enters the following information for each collection point: postal address, average collecting time, and average demand (in thermal containers). After implementing the algorithm using C programming, this is run and, in few seconds, it obtains optimal (or near-optimal) collection routes that specify the collection sequence for each vehicle. Different scenarios using various types of vehicles have been considered. Unless new collection points are added or problem parameters are changed substantially, routes need to be designed only once.

**Results:**

The two laboratories in this study previously planned routes manually for 43 and 74 collection points, respectively. These routes were covered by an external carrier company. With the implementation of this algorithm, the number of routes could be reduced from ten to seven in one laboratory and from twelve to nine in the other, which represents significant annual savings in transportation costs.

**Conclusions:**

The algorithm presented can be easily implemented in other laboratories that face this type of problem, and it is particularly interesting and useful as the number of collection points increases. The method designs blood collection routes with reduced costs that meet the time and capacity constraints of the problem.

## Background

One of the main challenges in healthcare systems today is to deliver high-quality services with limited resources. Therefore, optimization problems in healthcare have attracted the attention of many researchers, in particular from the area of Operations Research (OR). A survey on the application of OR in healthcare concludes that, although many healthcare problems have been successfully solved using OR techniques, many more still need the attention of researchers to provide effective and realistic solutions [1].

This paper presents a project to improve the logistics of blood sample collection at two important clinical laboratories in Catalonia, a region in the Northeast of Spain. Clinical laboratories perform blood analyses to gather information about the physical and chemical properties of blood. This information is essential for physicians to diagnose and manage certain diseases and conditions. One of the fundamental features of every clinical analysis refers to quality assurance. As a matter of fact, in 2003 (later revised in 2007) the International Organization for Standardization developed the ISO 15189 standard with the requirements for quality and competence in clinical laboratories. The standard involves the design of quality systems for the entire analytic process. This process consists of three phases: (1) a *pre-analytical* phase with analysis request, collection, transportation, and preparation; (2) an *intra-analytical* phase with testing; and (3) a *post-analytical* phase with results transmission, interpretation, and action. Special attention must be given to the pre-analytical phase, where most of the errors occur [2]. In the case of blood analyses, samples are usually collected daily from different collection points, such hospitals and health centers, and transported in thermal containers to a core laboratory for testing in the pre-analytical phase. Inadequate transportation and handling is a common pre-analytical error [3]. According to [4], blood must be carried to the laboratory under proper temperature (to preserve its properties), correct positioning (to avoid hemolysis) and within a given time window (less than two hours). Failing to do so may alter testing results, leading to misdiagnosis and inappropriate treatment. Blood transportation, therefore, poses a challenging logistics problem, where time is crucial to guarantee the quality of the samples.

### Laboratories of study

With the objective of improving healthcare delivery and bringing services closer to users, the Catalan Government established, some decades ago, a network of centers called *sample collection modules* made up of different facilities from where samples of blood and other clinical specimens could be collected for analysis. In recent years, to rationalize resources and search for economies of scale, there has been a centralization of testing processes. As a result, a small number of laboratories concentrate all analytic processes. This has lead to higher levels of efficiency while increasing the technical capabilities of these laboratories. One of such laboratory is Catlab, located in the Vallès Occidental region in Catalonia (Spain). Catlab was created after the merger of the Terrassa Health Consortium (“Consorci Sanitari de Terrassa”, CST) and the Terrassa Mutual Company (“Hospital Universitari Mútua Terrassa”, HUMT) to provide high quality and technological advanced clinical services. This central laboratory was located in the Logistics Park of Health in Viladecavalls, a municipality near Terrassa, the capital of the Vallès Occidental region. Catlab currently processes around 7.6 million clinical analyses annually, but has capacity to reach 11 million. With a 5.5 million euro investment in equipment and technology, it serves more than eight hundred thousand people in the region. Catlab receives blood samples daily from 43 collection modules dispersed across the Vallès Occidental region. These modules are clustered into four groups according to the company responsible for their management, namely, CST, HUMT, Sabadell Health Catalan Institute (ICS Sabadell), and Cerdanyola Health Catalan Institute (ICS Cerdanyola). Currently, each of the four companies schedules their collections and plans the routes independently, using a different carrier company. This is executed manually with few cost considerations, sometimes resulting in expensive daily deliveries.

The difficulties encountered by Catlab managers when designing collection routes motivated this joint work. The process was later replicated at another laboratory of the Doctor Robert Health Center (“CAP Doctor Robert”). This other laboratory, located in Badalona (a city in the Barcelona metropolitan area) concentrates all analytic processes from 74 surrounding collection modules.

## Methods

### The blood sample collection problem

The Blood Sample Collection Problem (BSCP) aims to find the routes to collect blood samples from different locations and to deliver them to a clinical laboratory for analysis. The BSCP is a variant of the well-known Capacitated Vehicle Routing Problem (VRP) [5] with two additional features: (i) routes are open, and (ii) time per route is constrained. In a regular VRP, a fleet of vehicles is based at a single depot to serve demands for a set of geographically dispersed customers. Each vehicle, whose capacity cannot be exceeded, leaves the depot, visits some customers, and returns to the depot. The problem consists in finding the sequence of deliveries (routes) so that all customers are served and the total distance traveled by all vehicles is minimized.

The VRP is a non-deterministic polynomial-time hard (NP-hard) problem [6], which implies a non-polynomial increase in the size of the solution space when the number of nodes is increased. Although significant research effort has been dedicated to the VRP, the problem still gets the attention of many researchers [7].

In a VRP, routes are closed in the sense that vehicles start and finish at the depot. In the BSCP, however, this is not the case since routes are covered by an external carrier company whose vehicles start at the first collection point, visit other collection points, and finish at the laboratory. This is called Capacitated Open VRP (COVRP) in OR literature and has also been studied extensively. The reader is referred to [8] for a review of solution methods proposed to solve the COVRP. The second distinctive feature in the BSCP is a time constraint imposed on the duration of each route. This time constraint is an upper bound determined by the maximum time that blood samples can last without deterioration, that is, two hours [9].

Considering these two additional constraints, the BSCP can be defined as a Capacitated Time-Constrained Open Vehicle Routing Problem (CTCOVRP). The capacity constraint is determined by the number of thermal containers that a vehicle can transport, whereas the time constraint is given by the two-hour time window between the first collection and delivery to the laboratory. The objective of the BSCP is to find a set of open routes to collect all blood samples within two hours that satisfies the vehicle-capacity constraint and minimizes total logistics costs. In the case of the laboratories studied in this paper, these costs derive primarily from the number of vehicles used by the external carrier company. Another possible objective function could include other distance- or time-related costs associated to these routes.

**Figure 1:**
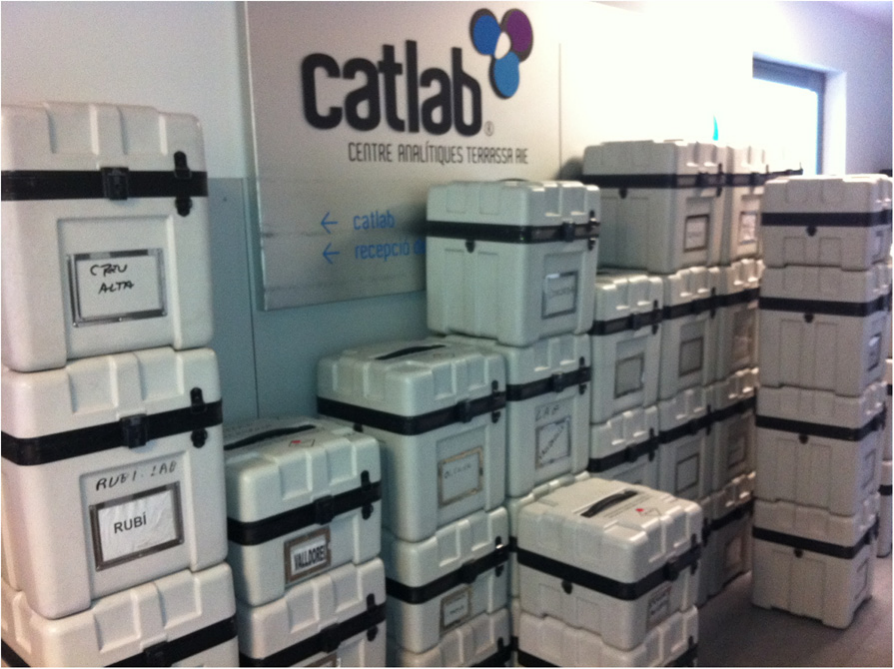
**Thermal containers.** Thermal containers to deliver blood samples to the laboratory.

Prior to solving the BSCP, the following input data is needed:

• Location of each collection point and the clinical laboratory;

• Travel distances and times between every pair of collection points, and between all collection points and the laboratory. This data was obtained with a web application that uses Google Maps developed by the authors;

• Average daily demand, in thermal containers (see Figure 1), for each collection point;

• Vehicle capacities, in thermal containers (i.e., 10, 16 or 25);

• Time constraint between the first collection point and laboratory (i.e., 2 hours);

• Average stopping time at each collection point: this time, which is usually between 10 and 15 minutes, consists in parking the vehicle at the center, filling up the corresponding forms, picking up the containers with blood samples, and loading them into the vehicle.

The CTCOVRP can therefore be described as the following optimization problem:

• Objective function: Minimize the number of vehicles.

Subject to:

• Feasible Routes: All routes start at a collection point and finish at the laboratory (i.e., Open VRP);

• Time Constraint: the time between collection at the first point and the delivery to the laboratory must be no greater than two hours;

• Capacity Constraint: total demand in thermal containers transported by a vehicle must be no greater than its maximum capacity (i.e., 10, 16 or 25).

Besides the BSCP, there exist other real applications of the CTCOVRP. For instance, a special version of the school bus problem known as the bus route generation problem [10]. Another application is found in the retailing industry, where many retailers outsource its distribution to third-party logistics providers that deliver goods from a depot to the stores without returning to the depot.

In the context of healthcare, and in particular, in the area of laboratory management, only one paper seems to describe a similar problem [11]. However, their authors only solve a small problem by complete enumeration, something infeasible in this study due to the large number of collection points (e.g., a case with 50 collection points would represent choosing routes among more than 3 × 10^68^ different possible combinations).

### Solution approach

The choice of a solution method or algorithm to solve an optimization problem of this type must consider both the solution quality (i.e., the cost of the solution) and the time to obtain it. Routing problems in general are difficult to solve in reasonable time. Therefore, heuristic methods are preferred since they are able to obtain excellent results in reduced time [7]. A heuristic is a computational method tailored to solve large optimization problems, like the one presented in this paper, known to be very complicated to solve optimally. Starting from an initial solution, a heuristic method searches iteratively for better solutions using a series of rules and conditions. Heuristics share many desirable features that prove to be excellent to solve complex problems: most of them are simple, easy to implement, robust and highly effective on difficult problems [12].

The algorithm designed to solve the BSCP is a heuristic based on a Genetic Algorithm (GA) [13]. GAs are robust and effective algorithms computationally simple and easy to implement. They generate new solutions using techniques inspired by natural evolution [14]. Each solution is obtained by decoding the chromosome of each individual. This chromosome has an associated fitness level correlated with the objective function. The GA produces a series of generations and the most fit individual of the last generation is the final solution. New generations are obtained by combining individuals of the current generation in a process called crossover. In some cases, random mutations of the individuals occur to explore new solutions.

**Figure 2:**
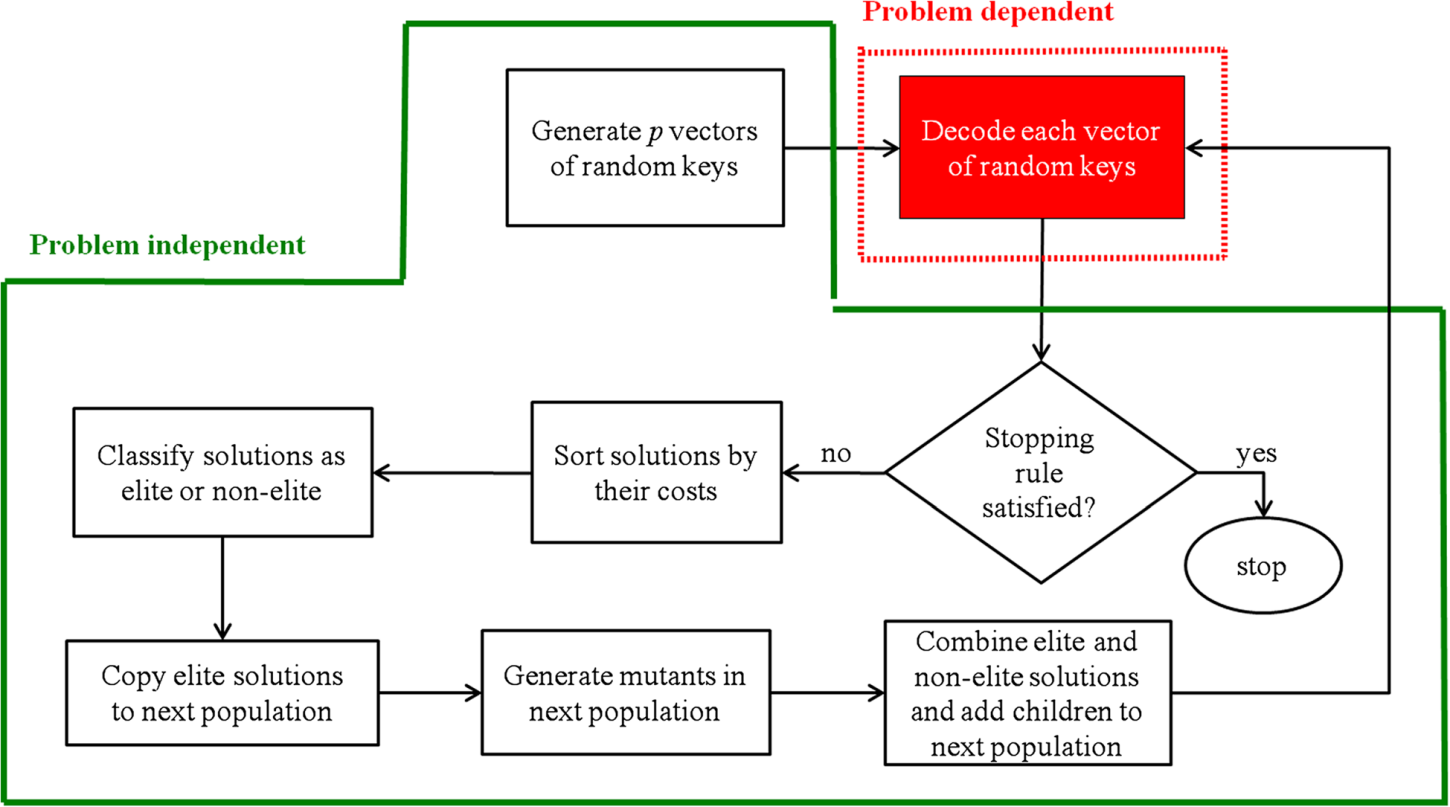
**Algorithm framework.** Framework for the genetic algorithm used to solve the BSCP.

The framework for the GA used to solve the BSCP is depicted in Figure 2. An individual’s chromosomes are represented by *n* random keys (genes) that are real-valued numbers in the interval [0,1]. This type of GA is known as Random Key Genetic Algorithm (RKGA), and it was first introduced to solve sequencing problems [14].

**Figure 3:**
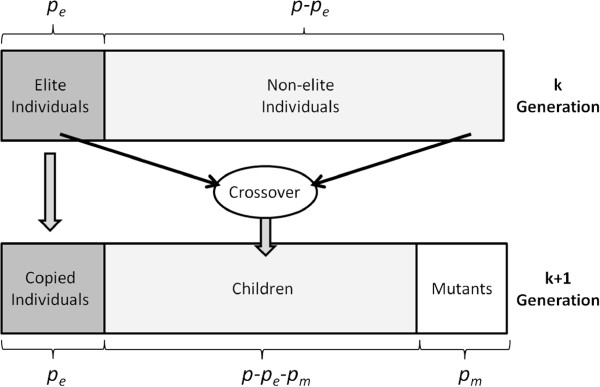
**New generation transition.** Transition of a new generation in the BRKGA.

In a RKGA, there is an initial population of *p* individuals, that is, *p* strings (or vectors) of *n* randomly generated numbers between 0 and 1. Using a deterministic algorithm, called decoder, the algorithm translates each individual’s random-key vector to obtain a corresponding feasible solution of the BSCP with its corresponding cost. This decoder, in other words, decodes solutions encoded as vectors of keys into feasible solutions for this problem. The population is then partitioned into two groups: a small group called the elite individuals with the *p*_*e*_ best individuals (around 10-20%), and a second group called the non-elite individuals, with the *p* - *p*_*e*_ remaining individuals (with *p*_*e*_ < *p* - *p*_*e*_). Next, this population is evolved to obtain the next generation. Figure 3 illustrates the transition of a new generation. First, all elite individuals are copied as they are. With this, the algorithm ensures that the best solutions are maintained in the population. Then *p*_*m*_ random individuals, called mutants, are added. Mutants are generated randomly like the individuals of the initial population. This operation, essential in GAs, enables the procedure to escape from local minima. Finally, the remainder of the population is composed of *p* - *p*_*e*_ - *p*_*m*_ additional individuals generated through the process of mating, or crossover. The original RKGA selects two parents randomly from the entire population. However, in this paper a variant called Biased Random Key Genetic Algorithm (BRKGA) [15], where one of the parents is always selected at random from the elite group of individuals, has been used. The other parent is selected at random from the non-elite population. Since *p*_*e*_ < *p* - *p*_*e*_, the probability of choosing an elite individual is larger than that of choosing a non-elite individual, that is, 1/*p*_*e*_ > 1/(*p* - *p*_*e*_). The given elite individual has then a higher likelihood to pass on its characteristics to future generations. The crossover, detailed below, will also contribute to this end. Repetition in the selection of a mate is allowed so that an individual may produce more than one offspring in the same generation.

**Figure 4:**
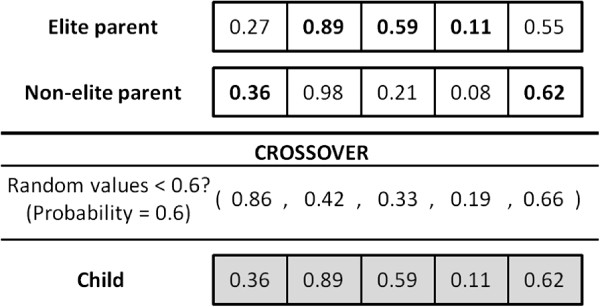
**Crossover example.** A crossover when the probability of choosing a gene from the elite parent is 0.6.

Mating is done using parameterized uniform crossover [16], that is, each random key (gene) of the child is chosen from one of its parents’ keys with a certain probability (defined by the user). The probability of inheriting the key of the elite parent must be larger than 0.5 to favor elite parent’s characteristics over the non-elite parent’s. Figure 4 shows a crossover when the probability of choosing a gene from the elite parent is 0.6. In the example, the new individual is obtained as follows. *n* (five in this case) random numbers between 0 and 1 are generated, one for each gene (that is, 0.86, 0.42, 0.33, 0.19 and 0.66). If the random number is below 0.6, the child inherits the gene of the elite parent; otherwise it inherits the gene of the non-elite parent.

When the next generation is complete with *p* individuals, these individuals are decoded into feasible routes and their costs are calculated, again using the decoder. This process is then repeated several times until a final solution is obtained. In practice, the algorithm stops when it reaches 100 generations without improvement. Increasing the running time will unlikely provide better results as shown in the Results Section. Note that a BRKGA can be used to solve a myriad of optimization problems; the only portion of the algorithm that needs to be adapted to each particular problem is the decoder.

The decoder for this vehicle routing problem is quite simple to obtain: each individual (solution) is composed of a string of real-valued numbers (random keys) in the interval [0,1]. These values are sorted obtaining a sequencing order. Routes are then obtained by cutting the sequence just at the point before problem constraints (capacity and time) are violated. For example, consider the child generated in the crossover in Figure 4 with the following random keys *p* = (0.36, 0.89, 0.59, 0.11, 0.62). The sequence obtained by sorting the keys of the chromosome is 4 - 1 - 3 - 5 - 2, which slightly differs from those of its parents (4 - 1 - 5 - 3 - 2 and 4 - 3 - 1 - 5 - 2, respectively). Suppose now that each point has one container to be delivered and the vehicle’s capacity is two containers. Therefore, three routes are obtained: 4-1-lab, 3-5-lab, and 2-lab. In the route construction, when cutting the sequence, both capacity and time constraints are considered. Since all collection points must be within two hours of the laboratory, the decoder always obtains feasible solutions from individuals.

## Results

The BRKGA has been implemented using the C programming language, and run on an Intel(R) Core(TM)2 T7500 with 2.2 GHz and 3 GB of RAM memory. For managerial reasons, the 43 collection modules were divided into two groups: a first group that includes all CST and HUMT centers (i.e., 18 centers); and a second group that includes all ICS Sabadell and ICS Cerdanyola centers (i.e., 25 centers). Two different scenarios were then considered when calculating collection routes:

I. Two separately calculated sets of routes, one for CST + HUMT centers and the other for ICS centers.

II. A jointly calculated set of routes for all 43 centers (CST + HUMT + ICS).

Each scenario was run twice assuming a vehicle capacity of 16 and 25 thermal containers, respectively. Table 1 shows both the current routes and the routes obtained after running the BRKGA until the stopping criterion was reached (that is, after 100 generations without a solution improvement). The algorithm was really fast in obtaining these routes, spending only between 1 and 27 seconds for the different scenarios. The current routes for the four companies are ten in total, distributed as follows: two routes for CST, three routes for HUMT, three routes for ICS Sabadell, and two routes for ICS Cerdanyola. These routes use vehicles with capacity of 16 and/or 25 thermal containers indistinctively.

**Table 1:** Number of routes for Catlab

		**Current solution**	**BRKGA solution**	
			**Vehicle capacity = 16**	**Vehicle capacity = 25**
Scenario I	CST + HUMT	5	3	3
	ICS	5	5	4
Scenario II	CST + HUMT + ICS	-	7	7

Number of routes for Catlab under Scenarios I and II using vehicles with two different capacities (16 and 25 thermal containers, respectively).

**Figure 5:**
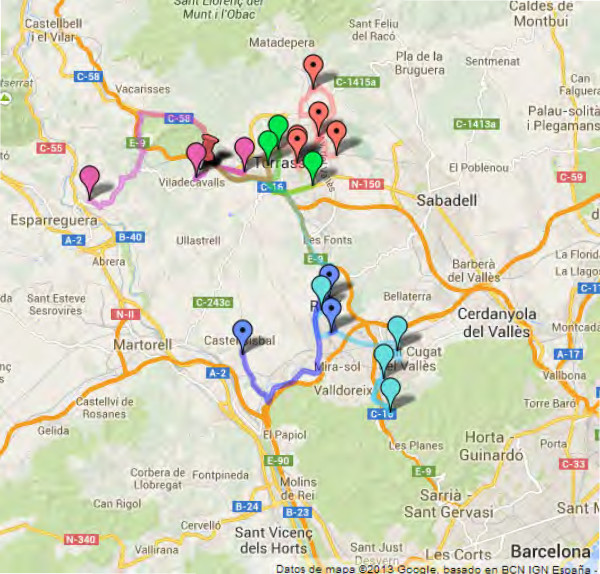
**Current routes for CST + HUMT scenario.** Current five routes used by Catlab for the collection centers of CST and HUMT. The pushpin represents Catlab’s central laboratory.

**Figure 6:**
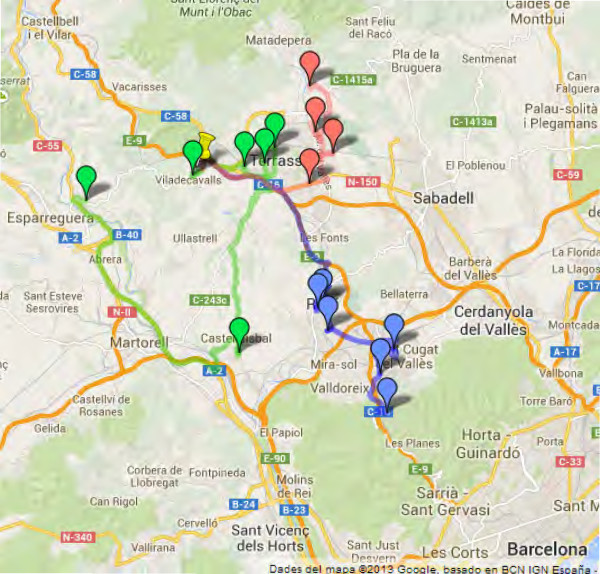
**Routes proposed by the BRKGA for CST + HUMT scenario.** The three routes proposed by the BRKGA for the collection centers of CST and HUMT. The pushpin represents Catlab’s central laboratory.

When the vehicle used to collect blood samples has a capacity of 16 thermal containers, the implementation of the algorithm reduces the number of routes from 10 to 8 for Scenario I and to 7 for Scenario II. Figure 5 maps the current 5 routes used by Catlab for CST + HUMT in Scenario I, while Figure 6 shows the solution proposed by the BRKGA using only 3 routes. When the vehicle has a capacity of 25 containers, on the other hand, the number of routes obtained is reduced to 7 for both scenarios.

To benchmark these results, the mathematical formulation for the CTCOVRP was implemented on a commercial solver (IBM ILOG CPLEX Optimizer 11.2) [17]. This type of software can handle relatively small problem instances using exact methods. As problem size increases, the computational time needed to solve a problem optimally grows exponentially, and CPLEX can only provide upper and lower bounds of the optimal solution. Table 2 shows the results given by CPLEX.

**Table 2:** Upper and lower bounds on the number of routes for Catlab using CPLEX

		**CPLEX solution**	
		**Vehicle capacity = 16**	**Vehicle capacity = 25**
Scenario I	CST + HUMT	4(3)	4(2)
	ICS	5*	6(3)
Scenario II	CST + HUMT + ICS	13(6.43)	14(5)

Upper (lower) bound obtained by CPLEX after running the program for 1 hour (Scenario I: CST + HUMT), 2 hours (Scenario I: ICS), and 3 hours (Scenario II: CST + HUMT + ICS), respectively. A number with an asterisk represents the optimal solution (i.e., upper and lower bounds coincide).

The BRKGA performs considerably well: when vehicle capacity is 16, it obtains optimal solutions in all cases. Note that in Scenario II, the lower bound found by CPLEX is non-integer (6.43) which implies that the optimal solution has at least 7 routes (it has actually 7 because the BRKGA solution is 7). When vehicles with capacity of 25 are used, the BRKGA provides high-quality solutions. Since these solutions are close to the lower bounds obtained by CPLEX, it is very likely that they are also optimal. The cost of using a vehicle is around €60 per route for 16-container vehicles, and €67 per route for 25-container vehicles. Reducing 3 routes every day, for a total of approximately 250 working days, implies annual savings in transportation of over €45,000, which represents 30% of the total annual transportation cost.

CAP Doctor Robert’s laboratory, on the other hand, collects blood samples from 74 collection modules. This collection is also carried out by an external carrier company, but unlike Catlab, they consider all collection points jointly when planning the routes. The route planning is done manually resulting in twelve different routes. In their case, they can use three types of vehicles with capacities 10, 16, and 25 thermal containers, respectively. Table 3 shows the results in number of routes. The BRKGA reduces the number of routes from 12 to 10 or 9 depending on the vehicle used. The improvement, in this case, represents savings of around 20% in transportation costs.

**Table 3:** Number of routes for CAP Doctor Robert

	**Current solution**	**BRKGA solution**		
		**Vehicle capacity = 10**	**Vehicle capacity = 16**	**Vehicle capacity = 25**
CAP Dr. Robert	12	10	9	9

Number of routes for CAP Doctor Robert using vehicles with three different capacities (10, 16 and 25 thermal containers, respectively).

## Discussion

Clinical laboratories provide essential public health services, obtaining invaluable information for physicians to prevent, diagnose and treat diseases. The demand for these services in particular, and for healthcare in general will continue to increase, and so will the costs, due to aging population and advances in medical knowledge and technology, among other factors [18]. Public resources for healthcare will remain insufficient to meet such increasing demand and costs. Therefore, policy makers, healthcare providers and, consequently, laboratory managers need to allocate limited resources efficiently to continue striving for excellence. An important part of the total laboratory expense derives from operational aspects in the daily activities of the laboratory. Their management and the problems encountered resemble the traditional manufacturing-related problems in Operations Management. Thus, the use of OR techniques, tools and theories can benefit substantially health care management. Many successful examples have been documented in literature [19-21], but there is still much potential for improvement in laboratory management.

The main benefits of applying OR methodologies, and, in this case, a solution method based on the BRKGA, are not only in terms of money savings in transportation. They also lead to a better management and decision making when problem circumstances change (e.g., the addition or modification of collection points), and to an improved quality service (e.g., by ensuring the two-hour time constraint on routes). Strategically, these methods are crucial for a better planning in case that a new laboratory merger had to be implemented due to the current economic turmoil.

The BRKGA is an algorithm that has been used successfully in numerous applications such as job-shop and project scheduling problems, assembly line balancing, tollbooth locations, etc. The core functioning of this approach is very similar regardless of the application since its architecture can be divided in a problem-independent component and a problem-specific part. This makes the algorithm really flexible: one only needs to set up few parameters (number of genes, size of population, percentage of elite individuals and mutants, and probability of inheriting elite genes), and construct a decoder that maps each random-key vector into a feasible solution for the problem being considered. This type of GA generally produces results that are as good as or better than those found using standard GAs [15].

## Conclusions

This paper has presented the operational problem of blood sample collection faced by two large laboratories, and used an advanced optimization technique to solve it. In particular, a Biased Random Key Genetic Algorithm has been implemented to find a set of collection routes that reduces approximately between 20% and 30% of the total logistics costs for two of the largest clinical laboratories in Spain. These routes, optimal in most cases, need to be calculated only once as long as the volume of samples to be collected in the different centers does not vary excessively, or new collection centers are not added.

This work is easily replicable to other laboratories that need to collect samples from different centers. A spreadsheet file with postal addresses, average demands and collecting times is the only data required to run the algorithm. Similarly, this model can also be adapted to other routing problems faced by clinical laboratories or health centers with slight changes in the constraints or the objective function. In general, the application of such OR techniques is particularly interesting and relevant as the problem size increases, since the difference between their solutions and those manually-obtained can be quite significant.

## Abbreviations

OR: Operations research; CST: Terrassa’s health consortium; HUMT: Terrassa’s mutual company; ICS: Health Catalan Institute; BSCP: Blood sample collection problem; VRP: Vehicle routing problem; NP: Non-deterministic polynomial-time; COVRP: Capacitated open vehicle routing problem; CTCOVRP: Capacitated time-constrained open vehicle routing problem; GA: Genetic algorithm; RKGA: Random key genetic algorithm; BRKGA: Biased random key genetic algorithm.

## Competing interests

The authors declare that they have no competing interests.

## Authors’ contributions

AG, HR, LSP and MGCR jointly designed the BRGKA applied to this problem context. IC and NB conducted the data gathering process. LSP ran the model and HR interpreted the results. AG led drafting and revising of the overall manuscript. All authors have read and approved the final version of the manuscript.

## Pre-publication history

The pre-publication history for this paper can be accessed here:

http://www.biomedcentral.com/1472-6963/14/12/prepub
